# A population-based cohort study examining the association of documented bladder diverticulum and bladder cancer risk in urology patients

**DOI:** 10.1371/journal.pone.0222875

**Published:** 2019-10-15

**Authors:** Chu-Wen Fang, Vivian Chia-Rong Hsieh, Steven Kuan-Hua Huang, I-Ju Tsai, Chih-Hsin Muo, Shih-Chi Wu

**Affiliations:** 1 Division of Urology, Department of Surgery, Chi Mei Medical Center, Tainan, Taiwan; 2 Department of Health Services Administration, China Medical University, Taichung, Taiwan; 3 Management Office for Health Data, China Medical University and Hospital, Taichung, Taiwan; 4 Graduate Institute of Clinical Medical Science, China Medical University, Taichung, Taiwan; 5 Trauma and Emergency Center, China Medical University Hospital, Taichung, Taiwan; National Yang-Ming University, TAIWAN

## Abstract

**Objectives:**

Studies have shown a high risk of tumor development within a bladder diverticulum (BD). We were interested in the relationship between BD and the development of bladder cancer. Herein, we attempted to investigate whether there exists an association between documented BD and subsequent risk of bladder cancer.

**Methods:**

We identified 10,662 hospitalized urology patients, including 2,134 documented BD patients (study cohort) and 8,528 non-BD subjects (comparison cohort) from Taiwan’s National Health Insurance database. Only urology patients were enrolled in the study to minimize selection bias. The two cohorts were frequency-matched 1:4 by age, sex and index-year. Patients with less than one year of follow-up were excluded to avoid inverting cause and effect. Risks of developing bladder cancer were estimated using the Cox proportional hazard regression model.

**Results:**

There was an increased bladder cancer risk in the documented BD patients. The incidence of bladder cancer in documented BD patients was 2.60-fold higher than that in the comparison group, and the overall risk-factor-adjusted hazard ratio was 2.63 (95% CI, 1.74–3.97). Moreover, stratified analysis by sex also showed that documented BD patients were at higher risk of subsequent bladder cancer than the comparison cohort. The effect of BD on the risk of bladder cancer was higher in males than in females and was more profound in patients without comorbidities than in those with comorbidities.

**Conclusion:**

In this population-based longitudinal study, urology patients with documented BD might have an elevated risk of subsequent bladder cancer. Based on the limitations of the retrospective study design, further studies are required.

## Introduction

Bladder cancer is the ninth most common malignancy worldwide and is the most common malignancy involving the urinary system [1], while urothelial carcinoma is the predominant histologic type around the world [2–3].

Risk factors for bladder cancer may include age, sex, smoking, diabetes, race, exposure to chemicals, chronic inflammation, and genetic factors [4]. In addition, the risk of tumor development within a bladder diverticulum (BD) has been suggested to be higher than that in the main bladder, which might be attributed to prolonged exposure to carcinogens within the intradiverticular mucosal lining [5].

BD is an outpouching of the bladder urothelium through the muscular wall of the bladder, creating a defect. This condition leads to ineffective emptying of urine due to a lack of muscle fibers, causing retention of urine and potential exposure to carcinogens [5–6]. However, there is a lack of direct symptoms and signs in BD patients. The diagnosis is often made incidentally and is often established by contrast images, echography, and cystoscopy [5].

Recent studies have observed various types of carcinoma arising from within BD [7–12]. However, there is still limited evidence regarding the link between BD and the development of bladder cancer. Therefore, we aimed to investigate whether there exists an association between documented BD and subsequent risk of bladder cancer in urology patients.

## Methods

### Data source

We used inpatient claims data from the Taiwan National Health Insurance Research Database (NHIRD) from 1996 to 2013. This database contains detailed medical histories of the hospitalized enrollees in Taiwan. The National Health Insurance (NHI) program is a universal health care system that was launched in 1995 with a current coverage of over 99%. The disease diagnoses were identified using the International Classification of Diseases, 9^th^ Revision, Clinical Modification (ICD-9-CM). Under the current system, a disease diagnosis without valid supporting clinical evidence may be considered medical fraud by the NHI program, potentially with a penalty of 100 times the payment claimed by the treating physician or hospital.

### Study subjects

We identified patients with new diagnoses of BD (ICD-9-CM 596.3) between 2000 and 2012 from the urology hospitalization records. To minimize selection bias, only urology patients were enrolled in the study. The index date for BD patients was defined as the date of BD admission. Patients less than 18 years of age or with malignancy (ICD-9-CM 140–208) before the index date were excluded from this study. Patients with less than one year of follow-up were also excluded to avoid inverting cause and effect. The comparison group was randomly selected from patients hospitalized in urology without a diagnosis or any documentation of BD. They were then frequency-matched to BD patients by age (exact year), sex and index year at a 1:4 case-to-control ratio. The exclusion criteria for the comparison group were the same as those for the BD group.

To comply with the Personal Information Protection Act for research use, all the identifying information of the insured people was removed and replaced with surrogate numbers. This study was approved by the Research Ethics Committee of China Medical University and Hospital in Taiwan [CMUH106-REC3-085].

### Outcomes and risk factors

The main outcome of interest in this study was the development of bladder cancer (ICD-9-CM 188) subsequent to the diagnosis of BD. All study subjects were followed from the index date to the occurrence of bladder cancer, withdrawal from the insurance program, or December 31, 2013, whichever came first. The comorbidities examined included diabetes (ICD-9-CM 250) [13–14], hydronephrosis (ICD-9-CM 591) [15–16], smoking-associated diseases (ICD-9-CM 305.1, 430–438, 410–414, 493, 496) [17–18], and chronic kidney disease (CKD) (ICD-9-CM 585) [19–20], which were extracted within two years before the index date and considered confounding factors. In addition, geographical regions of residence were also considered; we classified residential areas into four categories: northern, central, southern, and eastern Taiwan.

### Diagnosis of bladder cancer

In addition to the use of ICD-9 codes. In Taiwan, various cancer patients were precisely diagnosed by specialists based on pathology and clinical reports and were registered as “catastrophic illness patients”. Thus, we could define bladder cancer patients from this registry.

### Statistical analysis

Differences in demographics and comorbidities between the BD and comparison cohorts were examined using Chi-square tests for categorical variables and Student’s t-tests for continuous variables. Hazard ratios (HRs) and 95% confidence intervals (CIs) were calculated with Cox proportional hazard regression models. Proportional hazard assumption was measured by adding an interaction term between the study groups (BD/comparison group) and the logarithm of follow-up time in the Cox regression model. The hazard functions were proportional over time, and the assumptions for the Cox proportional model were not violated. Multivariate Cox regression models were adjusted for multiple variables that appeared to be statistically significant in the crude model. Kaplan-Meier estimates were used to plot the cumulative incidence. A log-rank test was used to detect the difference in incidence between the two groups. All statistical analyses were performed using SAS version 9.4 software (SAS Institute, Cary, NC, USA). The significance threshold was set at 0.05 for a two-tailed p-value.

## Results

**Fig 1:**
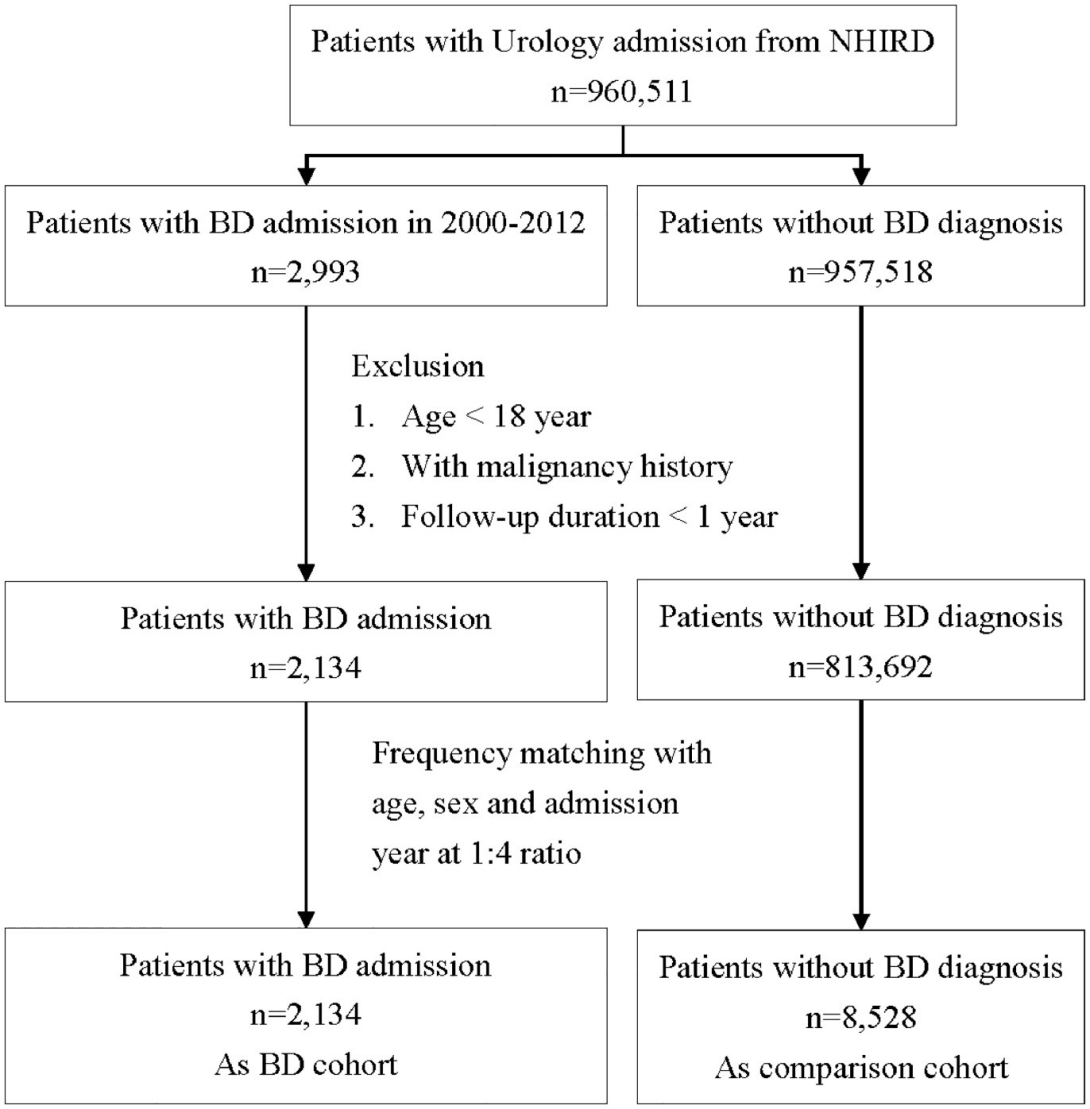
Flow chart of the study population.

We identified 10,662 hospitalized urology patients, including 2,134 documented BD patients (study cohort) and 8,528 non-BD subjects (comparison cohort) from Taiwan’s National Health Insurance database (Fig 1).

Distributions of age and sex were similar between the cohorts after matching. A total of 36.3% of BD patients lived in northern Taiwan, and 42.0% lived in southern Taiwan, while 42.5% of non-BD patients led in northern Taiwan, and 27.8% lived in southern Taiwan. Diabetes and hydronephrosis were more prevalent in non-BD patients than in BD patients (17.9% and 21.3%, respectively) (Table 1).

**Table 1:** Demographic and comorbidity characteristics of the BD and comparison cohorts.

	BD		Comparison		p-value
	N = 2134		N = 8528		
	n	%	n	%	
Age, year					0.98
18–64	597	28.0	2388	28.0	
> = 65	1537	72.0	6140	72.0	
Mean (SD)	70.3	(14.4)	70.2	(14.4)	0.93
Sex					0.99
Female	558	26.2	2230	26.2	
Male	1576	73.8	6298	73.8	
Region					<0.0001
North	775	36.3	3623	42.5	
Central	308	14.4	1938	22.7	
South	897	42.0	2367	27.8	
East	154	7.22	600	7.04	
Comorbidity					
Diabetes	305	14.3	1528	17.9	<0.0001
Hydronephrosis	269	12.6	1820	21.3	<0.0001
Smoking	463	21.7	1758	20.6	0.27
CKD	43	2.01	191	2.24	0.53

Chi-square test and t-test

BD: bladder diverticulum, CKD: chronic kidney disease

Overall, the incidence of bladder cancer in the BD cohort was 2.60-fold higher than that in the comparison cohort (3.17/1000 person-years for BD and 1.22/1000 person-years for the comparison cohort). The overall risk-factor-adjusted HR (aHR) was 2.63 (95% CI 1.74–3.97) after controlling for age and CKD (Table 2).

**Table 2:** Incidences and hazard ratios of bladder cancer based on a Cox proportional hazard regression.

				HR (95% CI]	
	Event no	Person-years	Incidence	Crude	Adjusted
BD					
No	58	47711	1.22	Ref.	Ref.
Yes	37	11674	3.17	2.60 (1.72-.3.93)***	2.63 (1.74–3.97)***
Age, year					
18–64	16	19483	0.82	Ref.	Ref.
> = 65	79	39901	1.98	2.42 (1.41–4.15)**	2.38 (1.39–4.08)**
Sex					
Female	19	15989	1.19	Ref.	
Male	76	43396	1.75	1.46 (0.88–2.42)	
Region					
North	36	24751	1.45	1.02 (0.58–1.80)	
Central	18	12643	1.42	Ref.	
South	34	17972	1.89	1.33 (0.75–2.35)	
East	7	4019	1.74	1.22 (0.51–2.92)	
Comorbidity					
Diabetes					
No	81	50834	1.59	Ref.	
Yes	14	8551	1.64	1.03 (0.58–1.81)	
Hydronephrosis					
No	81	47941	1.69	Ref.	
Yes	14	11444	1.22	0.72 (0.41–1.27)	
Smoking					
No	79	49098	1.61	Ref.	
Yes	16	10287	1.56	0.96 (0.56–1.65)	
CKD					
No	88	58450	1.51	Ref.	Ref.
Yes	7	935	7.48	5.02 (2.32–10.9)***	4.66 (2.15–10.1)***

Models adjusted for age, sex, area and comorbidities listed in Table 1

Incidence, per 1000 person-years

** p<0.01,

*** p < 0.001

BD: bladder diverticulum, CKD: chronic kidney disease

After stratification by age, sex, comorbidity and follow-up duration, the risk of bladder cancer in BD patients was significantly higher in all stratifications compared to control patients, except in the female group (Table 3). For sex-stratified analysis, the effect of BD on bladder cancer was higher in males than in females (interaction p<0.05). In the comorbidity-stratified analysis, the effect of BD on the risk of bladder cancer was also more profound among patients without comorbidity (aHR: 4.86, 95% CI 2.50–9.44) than among those with comorbidity (aHR: 1.78, 95% CI 1.04–3.04) (interaction p<0.05) (Table 3).

**Table 3:** Incidences and hazard ratios of bladder cancer based on a Cox proportional hazard regression stratified by age, sex, region, comorbidity, follow-up duration.

	BD		Comparison		HR (95% CI)	
	Event no	Incidence	Event no	Incidence	Crude	Adjusted
Age, year						
18–64	7	1.84	9	0.57	3.20 (1.19–8.60)*	3.33 (1.24–8.95)*
> = 65	30	3.82	49	1.53	2.49 (1.58–3.91)***	2.52 (1.60–3.96)***
Sex†						
Female	3	0.95	16	1.25	(0.22–2.61)	0.81 (0.24–2.79)
Male	34	3.99	42	1.20	3.31 (2.10–5.20)***	3.32 (2.11–5.22)***
Comorbidity status†						
No	17	4.01	18	0.88	4.52 (2.33–8.77)***	4.86 (2.50–9.44)***
Yes	20	2.69	40	1.47	1.83 (1.07–3.13)*	1.78 (1.04–3.04)*
Follow-up duration, years						
<5	25	3.03	44	1.32	2.30 (1.41–3.75)***	2.31 (1.42–3.78)***
≧5	12	3.51	14	0.97	3.57 (1.65–7.73)**	3.60 (1.66–7.78)**

Models adjusted for age and CKD

Incidence, per 1000 person-years

* p < 0.05,

** p<0.01,

*** p < 0.001

BD: bladder diverticulum

^†^Interaction p < 0.05

Because the association between bladder cancer and BD was different according to sex as previously seen in Table 3, we performed CKD-, benign prostatic hyperplasia (BPH), and transurethral resection of the prostate (TURP)-stratified analyses in males, as shown in Table 4. Consistent with our previous analyses, we found that BD patients still had an overall increased bladder cancer risk compared with non-BD patients in different CKD, BPH, and TURP stratifications (Table 4). The observed adjusted HRs appeared to be more statistically significant when we compared men among the non-comorbidity groups versus the comorbidity groups.

**Table 4:** Incidences and hazard ratios of bladder cancer based on a Cox proportional hazard regression stratified by CKD, BPH and TURP in men.

	BD			Comparison			HR (95% CI)	
	N	Event no	Incidence	N	Event no	Incidence	Crude	Adjusted
CKD								
No	1545	32	3.80	6174	40	1.16	3.27 (2.05–5.20)***	3.29 (2.07–5.24)***
Yes	31	2	17.86	124	2	4.30	4.06 (0.57–28.9)	4.01 (0.56–28.7)
BPH								
No	864	26	4.90	4013	29	1.17	4.21 (2.48–7.15)***	4.09 (2.41–6.95)***
Yes	712	8	2.49	2285	13	1.30	1.92 (0.79–4.63)	2.12 (0.87–5.13)
TURP								
No	954	20	3.99	3980	24	1.10	3.63 (2.00–6.57)***	3.56 (1.97–6.45)***
Yes	622	14	3.98	2318	18	1.37	2.90 (1.44–5.84)**	3.04 (1.51–6.12)**

Models adjusted for age and CKD

Incidence, per 1000 person-years

** p<0.01,

*** p < 0.001

BD: bladder diverticulum; CKD: chronic kidney disease; BPH: benign prostatic hyperplasia, TURP: transurethral resection of the prostate

**Fig 2:**
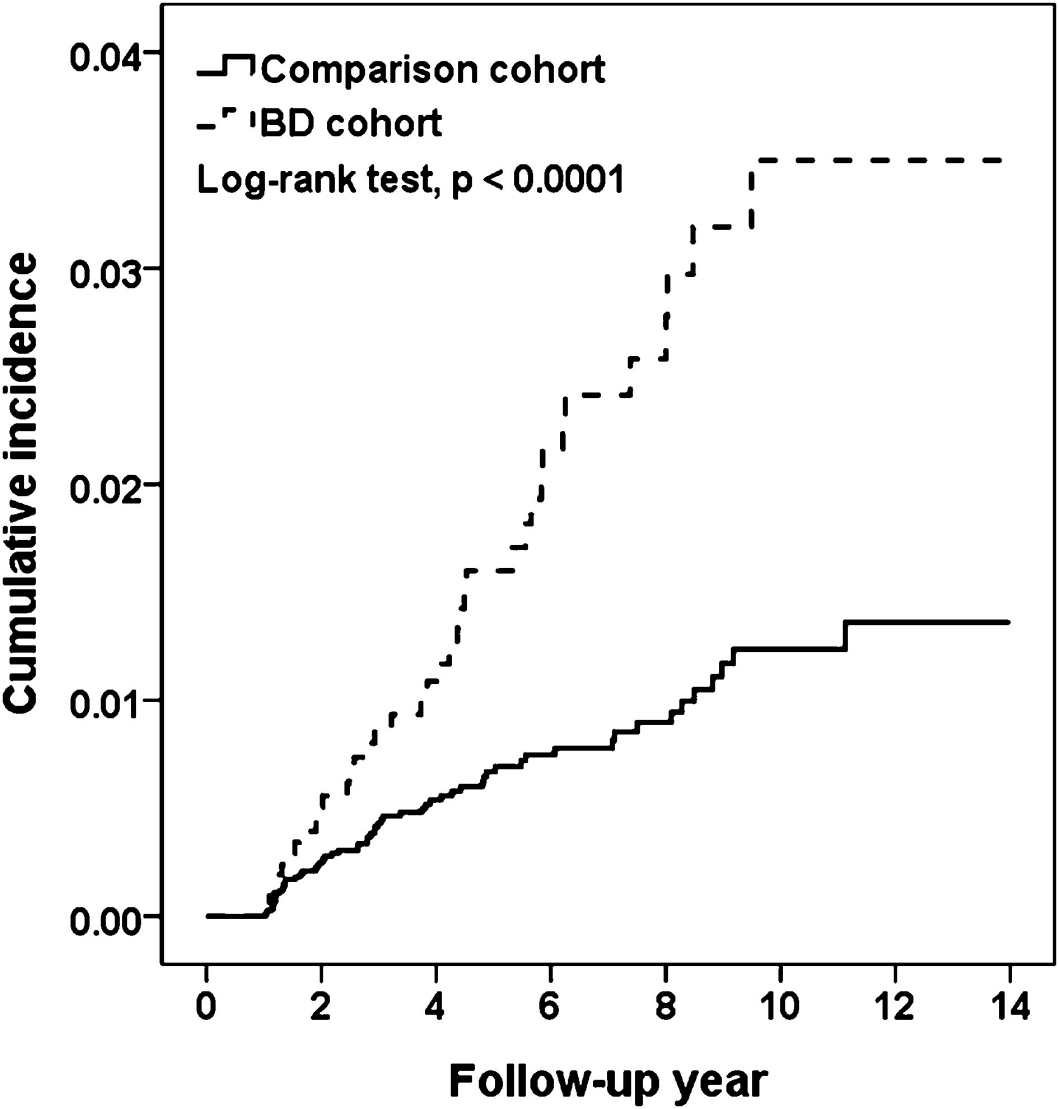
Cumulative incidences of bladder cancer in the comparison and BD cohorts.

Fig 2 shows that the cumulative incidence of bladder cancer in the BD cohort was 2.14% higher than that in the comparison cohort after a follow-up of 14 years (3.50% vs. 1.36%, respectively, log-rank test p<0.0001).

## Discussion

The development of a neoplasm within BD is an important issue because of its difficult diagnosis and early invasion secondary to a lack of musculature. The risk of developing a tumor within a bladder diverticulum is considered to be higher than that in the main bladder and may account for approximately 1% of all urinary bladder tumors.^5^

It has been reported that carcinogens in the urine accelerate the process of bladder carcinogenesis [21–22]. In addition, urinary hesitancy and residual urine retention may play some roles in the development of bladder cancer in males [21, 23].

Urinary stasis in BD might be associated with chronic infection and inflammation that lead to stone formation, dysplasia development, squamous cell metaplasia and leukoplakia, which increase the possibility of intradiverticular neoplasm development [24]. In addition, types of carcinoma that arise from BD include squamous cell carcinoma [7–8], adenocarcinoma [9], urothelial carcinoma [10], small cell carcinoma [11], and primary osteosarcoma [12]. Among these malignancies, urothelial carcinoma is the most common (78%), followed by squamous cell carcinoma (17%), a combination of transitional and squamous cell types (2%), and adenocarcinoma (2%) [25].

In our study, the overall adjusted hazard ratio for bladder cancer was high in BD patients compared to non-BD subjects (HR = 2.63). Given that there were a lack of intradiverticular residual urine volume data in BD patients, BDs might be effectively drained in some patients after micturition. It is plausible that this result might, at least in part, support the idea that urinary stasis in BD is associated with chronic inflammation and could potentially lead to the development of malignancy. In addition, the hazard ratio for bladder cancer was higher in patients aged more than 65 years (HR = 2.34 Table 2), which is consistent with the notion that there is a close relationship between aging and cancer [26–27].

In the current study, the prevalence of CKD was approximately 2% (Table 1), which is not consistent with other Taiwanese studies (6.9~11.9%) [28–31]. However, there were different sources and methods of selecting CKD patients among these studies. For instance, patients aged ≥20 years were selected from a community-based multiple screening program in one study [28], while another study selected patients from a standard medical program run by a private firm [29], and another study used the diagnostic code for CKD from inpatient or outpatient services to select patients [30]. In the current study, we selected urology patients from an inpatient rather than an outpatient data file, which may have accounted for the lower prevalence of CKD than that found in other Taiwanese studies.

There is a close relationship between CKD and bladder cancer [20, 32], and BPH has been reported to be a risk factor for bladder cancer in males [33]. However, in the current study, there was an increased incidence of bladder cancer in CKD patients (HR = 4.46. Table 2). After CKD- and BPH-stratified analyses in male patients, the BD cohort still had a higher bladder cancer risk than the comparison cohort with different CKD and BPH stratifications (Table 4). This result indicates that BD might be a risk factor for the development of bladder cancer in urology patients.

Early diagnosis of BD is challenging, and the prognosis of bladder diverticulum cancer can be poor [34–36]. The reported incidence of BD ranges from 1.7% to 13% [34, 37–39]. A recent cadaver study showed a higher rate of up to 23.4% [40]. This suggests that there are asymptomatic cases of BD. However, the prevalence of BD is low (0.31%, 2,993/960,511) in the current study. This could be attributed to lack of necessary examination or procedures to confirm a diagnosis of BD in the study population. Moreover, BD seems not to be a very important coding system in clinical urological practice in Taiwan. Thus, the coding of BD might not always be added by urologists since there are usually other major problems present, such as urinary tract infection, BPH, or other obstructive uropathy. Together, result in a probability of undocumented BD and a low prevalence of BD in our results. Therefore, our results cannot be generalized to all BD patients; however, they may be relevant to urology patients with documented BD.

Because there was lack of information on the location, severity, and specific identification of intradiverticular or intravesical bladder tumors in BD patients in the current data, it is difficult to confirm that whether the bladder cancer is developed from a BD. Therefore, our results are only representative of an overall elevated incidence of bladder cancer in urology patients with documented BD. The findings are also difficult to relate to the association between BD and the development of intradiverticular tumors. Owing to few data regarding the pathogenesis in this issue, we assume that urinary stasis and residual urine retention in BD may play some roles in the development of bladder cancer [21–23]. Therefore, further studies are required to elucidate this concern. However, our findings may suggest the need for a regular monitoring protocol in urology patients with documented BD.

To the best of our knowledge, no study has ever longitudinally examined the association between documented BD and the development of bladder cancer. In conclusion, the present large cohort study may reveal an association between documented BD and subsequent risk of bladder cancer in urology patients. This finding might partially be attributed to prolonged contact of urinary carcinogens due to urinary stasis. Future studies are warranted to investigate this proposed relationship and the underlying mechanisms.

### Limitations of the study

Our study has strengths of including a large study population, longitudinal design, reliable diagnosis and high follow-up rate, as well as subgroup analyses of the bladder cancer risks in urology patients with documented BD. However, certain limitations exist. First, the documented BD cases were based on admission diagnoses and codes. The information for location and severity, residual urine amount, size of BD, and accurate diagnosis of intradiverticular or intravesical bladder tumors was lacking. In addition, there were no pathology reports or stage/grade information for bladder cancer. Second, several factors, such as lifestyle (e.g., smoking, drinking), socioeconomic status, nutrition, and genetic factors, were not available for adjusting the risk of bladder cancer development. Third, relevant clinical variables (e.g., imaging results, histological findings, and laboratory data) were also unavailable because the data used were anonymous. Fourth, since urology patients with documented BD may have a higher likelihood of being diagnosed with bladder cancer during their follow-ups, detection biases do exist. However, this bias was minimized with the use of a washout period for analysis during the study period. Finally, despite our meticulous study design and control of confounding factors, there were still biases that resulted from the retrospective study design.

## Conclusion

In this long-term population cohort study, urology patients with documented BD might have an elevated risk of subsequent bladder cancer, which might suggest the need for a regular monitoring protocol in these patients. However, further studies are required to investigate the underlying mechanisms.

## Abbreviations

aHR: adjusted hazard ratio
BD: Bladder diverticulum
BPH: Benign prostatic hyperplasia
CI: Confidence intervals
CKD: Chronic kidney disease
HR: Hazard ratio
ICD-9-CM: International Classification of Diseases, 9^th^ Revision, Clinical Modification
NHI: National Health Insurance
NHIRD: National Health Insurance Research Database
TURP: Transurethral resection of the prostate
